# Literary Notes

**Published:** 1902-06

**Authors:** 


### LITERARY NOTES.

The Palisade Manufacturing Company, of Yonkers, has issued a syllabus of bacteriology which is designed to aid in the microscopic diagnosis of disease. It is a well-printed brochure of about 50 pages, and contains some fine plates in colors giving accurate appearances of the different bacilli of commoner existence. The directions for preparing sections for staining and making the necessary solutions therefor, together with the descriptions of the several bacilli are concise and based on recent studies. The title-page contains pictures of Koch, Pasteur, and Sternberg, an appropriate recognition of these great investigators. The publishers present it to physicians as "an accurate, succinct and yet comprehensive working guide" to enable them to conduct the ordinary and essential bacteriological examinations. It will be furnished upon application.

---

THE First Report of the New York State Hospital for the care of Crippled and Deformed Children, covering a period of ten months, ending September 30, 1901, has been issued. The hospital is located at Tarrytown and the excellence of its work is beyond dispute. The report contains a number of half-tone engravings, showing the characteristic features of the service. The consultants from Buffalo are Dr. Roswell Park and Dr. Charles G. Stockton.

---

BATTLE & CO., of St. Louis, have issued chart No. 7 of the surgico-anatomical series, illustrating fractures of the clavicle. The bony and muscular structures involved are graphically depicted, the colors and shades giving effective contrast to the injury. The indications for treatment are summarised in the description that accompanies the engraving.

---

THE Grosvenor Public Library, of Buffalo, has issued recently a catalogue of poetry in the English language, which the library contains. It is a brochure of 128 pages, imperial 8vo, and enumerates 3,542 volumes and 296 pamphlets. It will prove a valuable aid to all interested in poetry.

---

MONSIEUR A. MARIANI, of Paris, has lately published a brochure entitled, Eminent Physicians' Portraits and Biographical Sketches, compiled from Album Mariani, which has been noticed heretofore in these columns. This compilation contains woodcut engravings, beautifully executed, of thirty prominent members of the Paris Academy of Medicine, all of whom speak in praise of Vin Mariani. The pamphlet is furnished from the New York office, 52 W. Fifteenth Street.

---

THE Forty-third Annual Report of the Buffalo General Hospital, the same being for the year 1901, has been issued. The statistics of the year are: treated in the hospital, 2,852 cases, divided as follows—medical, 1,176; general surgical, 1,231; ophthalmological, 30; otological and rhinological, 83; gynecological, 332. Operations performed, 1,232, of which 856 were general surgical; 285 gynecological; 18 ophthalmological; 73 otological and rhino-laryngological. Total number of ambulance calls, 935.

*The Optical News* is the name of a new monthly leaflet, published by Mr. J. W. Jarvis, successor to the Fox Optical Co. Besides a number of interesting notes relating to spectacles and eye-glasses, the issue for May contains a notice of the removal of Mr. Jarvis's entire plant to 508 Main Street, Buffalo, where spacious and well-equipped rooms may be seen.

---

E. P. DUTTON & COMPANY publish an important book, entitled Sanatoria for Consumptives, which is a critical and detailed description, together with an exposition of the open-air or hygienic treatment of phthisis, by F. Rufenacht Walters, Fellow of the Royal College of Surgeons. Sir Richard Douglas Powell has written an introduction. The book describes the more important institutions, and in this way an account of the most approved methods of the treatment of consumption.

